# Anesthetic Agents in Operations for Gunshot Wounds

**Published:** 1852-09

**Authors:** John B. Porter

**Affiliations:** Surgeon, U. States Army


*Anesthetic Agents in Operations for Gunshot Wounds.* By John B. Porter, M. D., Surgeon, U. States Army.—In our former paper, the case of Williamson was presented with some remarks in relation to the use of sulphuric ether for producing anæsthesia in operations in the General Hospital at Vera Cruz, in 1847. In the summer of that year, an amputation of the thigh was performed, the patient having been put under the influence of ether, in which the hemorrhage was almost uncontrollable. The blood spouted in all directions, and I have never seen an operation where it was necessary to secure so many bleeding vessels. Even after every small vessel that could be got at was secured, it was necessary to use cold water freely to suppress the general oozing of blood. At the time, I imputed the obstinate hemorrhage to the pernicious influence of the ether. In gunshot wounds anæsthetic agents are almost universally unnecessary, and are almost universally injurious. It was for this reason that they were entirely given up in the hospital at Vera Cruz.

It may be well questioned whether anæsthetics are not calculated to produce injurious effects in all important amputations; but they certainly do so in operations performed for gunshot wounds. M. Velpeau says: "Chloroform evidently depresses the nervous system, and as great prostration always exists in patients who have received gunshot wounds, it is advisable to refrain from any anæsthetic means."—*Ranking's Abstract*, 1848. Mr. Alcock refers to the cases of soldiers wounded in battle, where the excitement is such as to carry them through almost any operation. I regret that Mr. Alcock's paper is not before me. These are the cases spoken of by Mr. Guthrie:

"Soldiers in general are anxious to undergo an operation when they find it inevitable, and frequently press it before the proper time; that is, before they have sufficiently recovered the shock of the injury."—*Gunshot Wounds*, p. 232. These are the cases which require a little more time, some "encouraging words," and perhaps a little wine or brandy and water; but no anæsthetics, for the patients are already sufficiently depressed.

There are two sets of cases; in the one (Velpeau's,) the shock to the nervous system is great, from which the patient may not recover, and the use of anæsthetics would be awfully destructive; in the other class, they are unnecessary, and would prove useless and injurious. In the flap operation, they must prove more injurious than in the circular; from the fact that muscle forms almost the entire covering for the stump; and the contractility of the muscular tissue is for a time almost annihilated, to be recovered irregularly at irregular intervals. Further, after the use of these agents wounds do not heal so readily by the first intention.

M. Jobert, on the use of ether, states that the local inflammation has proved less, and that union by the first intention has been prevented. I am able to bear testimony to the correctness of M. Jobert's statement.—*American Journal.*

